# Importance of team to increase compliance in adolescent spinal deformities brace treatment: a cross-sectional study of two different settings

**DOI:** 10.1186/1748-7161-7-S1-O5

**Published:** 2012-01-27

**Authors:** F Tessadri, A Pellegrini, M Tavernaro, A Zonta, S Negrini

**Affiliations:** 1 Orthotecnica, Trento, Italy; 2 ISICO, Milan, Italy

## Background

SOSORT Brace Treatment Management Guidelines highlight team’s role [1,2].

## Purpose

To verify the importance of rehabilitation team in adolescent patients bracing.

## Materials and methods

Population: 38 patients (28% hyperkyphosis, 72% AIS) extracted from one single CPO database; same MD; brace wearing for at least 6 months between 01/01/2008 and 01/09/2009; age 10 or more.

Methods: Two questionnaires: the SRS-22 [3,4], and one especially developed (QT) with 25 multiple choice questions about adherence to treatment (sections: brace, exercises, team).

Groups: the differences between the two PT teams were team building (G1 highly structured and collaborative) and setting (G1 private, G2 health national service). G1 included 13 patients and G2 25.

## Results

No population differences at baseline. Response rates: 92% QT and 69% SRS-22 (G1), 60% and 56% (G2) respectively. There was more compliance in G1 than in G2: in particular, brace wearing (75% vs 55%), exercises adherence (58,4% vs 36,3%), and social activities (92% vs 66%). In G1 there was less sport activities give up (0% vs 36,3%) and pain (7% vs 41%) than in G2. All domains of SRS-22 were strikingly different: function (4.13 G1 vs 2.90 G2), pain (3.93 vs 2.87), body image (3.84 vs 2.59), mental health (4.13 vs 2.91), satisfaction with treatment (4.17 vs 3.85). G1 had better radiographic results (6.7° improvement vs 4.2°).

## Conclusions

With the same MD and CPO (i.e. same brace and treatment type and quality), PT team building and setting plays a major role in compliance and final results.

## References

[B1] Negrini S Atanasio S Fusco C Zaina F Effectiveness of complete conservative treatment for adolescent idiopathic scoliosis (bracing and exercises) based on SOSORT management criteria: results according to the SRS criteria for bracing studies - SOSORT Award 2009 Winner Scoliosis 2009 4 19 10.1186/1748-7161-4-19 19732429 PMC3224944

[B2] Negrini S Grivas TB Kotwicki T Rigo M Zaina F Guidelines on "Standard of management of idiopathic scoliosis with corrective braces in everyday clinics and in clinical research": SOSORT Consensus 2008 Scoliosis 2009 4 1 2 19149877 10.1186/1748-7161-4-2 PMC2651850

[B3] Monticone M Baiardi P Calabrò D Calabrò F Forti C Development of the Italian version of the revised Scoliosis Reseacrh Society-22 Patient Questionnaire, SRS-22r-I: cross-cultural adaptation, factor analysis,reliability, and validity Spine 2010 35 24 E1412 1417 10.1097/BRS.0b013e3181e88981 21030889

[B4] Monticone M Carabalona R Negrini S Reliability of the Scoliosis Research Society-22 Patient Questionnaire (Italian version) in mild adolescent vertebral deformities Eura Medicophys 2004 40 3 191 197 16172587

